# A Comparison of the Force‐Velocity Relationship of Bonobo and Human Muscle Fibers

**DOI:** 10.1002/jez.70015

**Published:** 2025-07-22

**Authors:** Hans Degens, Maarten Bobbert, Melanie Scholz

**Affiliations:** ^1^ Department of Life Sciences, Institute of Sport Manchester Metropolitan University Manchester UK; ^2^ Institute of Sport Science and Innovations Lithuanian Sports University Kaunas Lithuania; ^3^ Faculty of Behavioural and Movement Sciences Vrije University Amsterdam the Netherlands; ^4^ Kinesiology and Health Sciences University of Waterloo Waterloo Ontario Canada

**Keywords:** curvature of the force‐velocity relationship, muscle function, power, primate, single fibers, specific tension

## Abstract

It has been reported that the muscles of chimpanzees and bonobos have “super strength” and it has been suggested that this is attributable to a larger specific tension and specific power of their muscles. To investigate this we compared the force‐velocity relationship in 85 human and 49 bonobo (*Pan paniscus*) skinned fibers at 15°C. Fibers were classified as type I or II with gel electrophoresis. Type II fibers had a higher maximal shortening velocity (Vmax) and lower curvature of the force‐velocity relationship (higher a/Po) than type I fibers in both species (*p* < 0.001). Although bonobo fibers of both types were larger and produced more force than human fibers, their specific tension and Vmax were lower (*p* < 0.001). The a/Po was higher in bonobo fibers (*p* < 0.001). Combined these differences in the parameters of the force‐velocity relationship resulted in a similar specific power in bonobo and human fibers. The lesser curvature of the force‐velocity relationship offsets the negative effects of a lower specific tension and Vmax on specific power of bonobo muscle fibers. The “super strength” of bonobos cannot be explained by differences in muscle fiber contractile properties but may reflect a higher proportion of type II fibers than in human muscle.

## Summary

The “super strength” of bonobos cannot be explained by differences in muscle fiber contractile properties but may reflect a higher proportion of type II fibers than in human muscle.

## 1 Introduction

During an unexpected encounter with a male chimpanzee (*Pan troglodytes, Eliotti*) in the Budongo Forest in Northern Uganda the startled chimp displayed behavior that led Alan Walker to comment that he “*came to appreciate firsthand … that great apes are immensely strong*” (Walker 2009). This subjective experience corresponds with observations in the 1920s that chimpanzees have stronger muscles than student members of the college football team who were “*well developed muscularly*” (Bauman 1926). A later study, however, found no difference in the absolute strength of chimpanzees and humans (Finch 1943). Nevertheless, both these and other studies agree that muscles from chimpanzees (Bauman 1926; Finch 1943; O'Neill et al. 2017) pound for pound muscle mass outperformed human muscles in terms of force and power generation. The same is true for bonobos (*Pan paniscus, Schwarz*), or pygmy chimpanzees (Scholz et al. 2006).

Several explanations have been suggested for this “super strength” of the chimpanzee and bonobo (Walker 2009). One suggestion is that chimpanzees are better able to recruit their muscles, perhaps because of a lower inhibition of muscle recruitment (Walker 2009). However, humans have been shown to be able to recruit at least 90% of their muscle (Erskine et al. 2009), hence a lesser ability to recruit muscles is unlikely and insufficient to explain the 50% higher muscle force or power in chimpanzees than humans (O'Neill et al. 2017).

Another explanation is that a higher power per unit muscle volume (specific power) in bonobo than human muscles is due to a higher maximal force per unit cross‐sectional area (specific tension) (Scholz et al. 2006). Yet, a comparison of the specific tension and unloaded shortening velocity showed no intrinsic differences in the muscle fiber contractile properties between humans and chimpanzees (O'Neill et al. 2017). As the specific power of type II fibers is about 4x that of type I fibers (Bottinelli et al. 1996; Gilliver et al. 2009) it was suggested that the higher performance of chimpanzee than human muscles may reflect a higher proportion of type II fibers (O'Neill et al. 2017).

Hitherto, it has not been established whether there are differences in the curvature of the force‐velocity relationship of fibers of a given type between humans and chimpanzees. This may well be significant as a lower curvature (higher “shape factor” or a/Po)—with specific tension and maximal shortening velocity (Vmax) being the same—will be associated with a higher power generating capacity (Gilliver et al. 2009). A higher a/Po will also mean that a muscle (fiber) can produce a higher force at a given proportion of Vmax. This, in fact, has been a proposed explanation of the higher jumping performance of bonobos than humans, where in bonobos “*the force delivered at a certain shortening velocity must have been very high*” (Scholz et al. 2006).

Although a study on marmosets did not comment on a/Po, the fibers of the marmoset (*Callithrix jacchus*) did have a higher a/Po that must have contributed to a higher power output in marmoset than human muscle (Plas et al. 2015). Based on these observations in the marmoset and the similar maximal unloaded shortening velocity and specific tension in human and chimpanzee muscle fibers (O'Neill et al. 2017), we hypothesized that at least part of the higher performance of bonobo muscles is related to a lower curvature (higher a/Po) of the force‐velocity relationship of bonobo compared to human muscle fibers.

## 2 Materials and Methods

The data from human single fibers (*n* = 85) from the vastus lateralis muscle from 18‐ to 25‐year‐old men have been published previously (Gilliver et al. 2009), but are included for comparison with the contractile properties of bonobo fibers (*n* = 49). The study was approved by the Manchester Metropolitan University Ethics Committee, and participants provided written informed consent before the biopsy was taken. The study adhered to the Declaration of Helsinki. The data from these human fibers were collected during the same period, with the same equipment and solutions, and by the same investigators as the bonobo fibers.

Opportunistic sampling of muscle biopsies from *mm. vastus lateralis* (VL; 26 fibers), *biceps brachii* (BB; four fibers), *soleus* (SOL; one fiber), *adductor magnus* (ADD; nine fibers) and hamstring (HAM; eight fibers) from one female bonobo (070124) was performed within 2 h after natural death from heart failure in de Apenheul, Apeldoorn, The Netherlands. For postmortem procedures on surplus tissues no animal research permit is required according to the Dutch Law on Animal Research in full compliance with EU regulations. The biopsies were collected, divided into small bundles in glycerol relax solution and stored for 24 h at 4°C. Then they were transferred to −20°C until use within 1 month.

The solutions were as described previously (Gilliver et al. 2009). The Relax solution contained 4.5 mM MgATP, 1 mM free Mg^2+^, 10 mM imidazole, 2 mM EGTA and 100 mM KCl. The low‐EGTA solution was the same as the relax solution, but 5 mM EGTA. The activating solution had a pCa4.5, 5.3 mM MgATP, 1 mM free Mg^2+^; 7 mM EGTA, 19.6 mM creatine phosphate and 64 mM KCl. All solutions were set at pH 7.0 with KOH.

At the day of the experiment a bundle was transferred for 20 min into relax solution with 1% Triton X‐100 on ice to further permeabilize the membrane. For the rest of the day, the bundle was kept on ice in relax solution. Single fiber fragments were isolated and attached to a force transducer (Aurora 403 A, Aurora Scientific Inc, Aurora, Ontario, Canada) and a motor arm (Aurora 312 C) of the permeabilized fiber system (Aurora 400) mounted on an inverted microscope (Olympus IX71). The system was kept at 15°C.

The sarcomere length was set at 2.53–2.69 µm using Fourier transformation of the sarcomere pattern (Aurora 900 A). This sarcomere length is around optimal length in both human and marmoset fibers (Plas et al. 2015) and most likely also applies to bonobo fibers. After setting the sarcomere length, the fiber cross‐sectional area (FCSA) was calculated from the diameter while the fiber was briefly suspended in the air, assuming the fiber had a circular circumference. The fiber length (FL) ranged from 0.70 to 3.97 mm. Then the fiber was transferred to a low EGTA solution for 15 s before being placed in the activating solution (pCa4.5). After the maximal force (Po in µN) was reached, the fiber was subjected to four series of four isotonic releases, where in each series the fiber did not shorten more than 20%. After completion of a series of four releases the fiber was rapidly restretched in pCa 4.5 to its original length, a procedure that has been shown to help stabilize the striation pattern (Brenner 1983). After completion of the contractile measurements the fibers were collected in Laemli sample buffer for subsequent determination of fiber type with poly‐acrylamide gel electrophoresis.

As described before (Gilliver et al. 2009), the shortening velocity at each isotonic release was determined from a least‐squares linear regression to the last 100 ms of a 150‐ms release step. The force and velocity data were fitted to the Hill equation (Hill 1938) using a nonlinear least‐squares regression iteration (Solver, Microsoft Excel) to give the best‐fit for the Hill constants “a” and “b.” The Hill equation was then used to estimate the maximal shortening velocity (Vmax in fiber lengths·s^‐1^ (FL·s^‐1^)). The specific tension was calculated as Po per unit cross‐sectional area (N·cm^‐2^), and a/Po is a measure of the curvature of the force‐velocity relationship (a low a/Po indicates a large curvature). The power‐force curve was used to calculate maximal power (µW) and specific power (W·Litre^‐1^).

Data from the isotonic releases were rejected if the Po dropped more than 10%, sarcomere length changed more than 0.15 µm and/or the R^2^ for the fit to the Hill equation was less than 0.96.

### 2.1 Statistics

Differences in the contractile properties between human and bonobo, and type I and type II fibers were determined with a 2‐way ANOVA in SPSS (28.0.0.0). Normality of the data was assessed with visual inspection of normality plots. If a significant interaction was found, Bonferroni‐corrected post‐hoc tests were performed to locate the differences. To assess the determinants of the specific power of muscle fibers, we performed a stepwise linear regression and chose the following variables: species, fiber type, specific tension, Vmax and a/Po. Variables were only included in the regression if their contribution significantly (*p* < 0.10) improved the R^2^_adj_. Differences were considered significant at *p* < 0.05 and data are presented as mean ± SEM.

## 3 Results

In the bonobo, there were no significant differences in contractile properties between different muscles, except for a smaller FCSA and Po in the ADD than the VL (*p* ≤ 0.034; Table 1).

**Table 1:** Contractile properties of fibers from different muscles from the bonobo.

	Type	FCSA (µm^2^)	Po (µN)	ST (N∙cm^‐2^)	Vmax (FL∙s^‐1^)	a/Po	Power (µW)	Power_spec_ (W ∙ L^‐1^)
VL	I (9)	9660 ± 1054	848 ±43	9.60 ± 1.14	0.256 ± 0.028	0.112 ± 0.012	0.024 ± 0.004	1.70 ± 0.34
	II (18)	10,164 ± 829	1273 ± 92	12.98 ± 0.66	0.794 ± 0.035	0.194 ± 0.009	0.145 ± 0.021	8.25 ± 0.63
BB	I (1)	5027	599	11.9	0.333	0.100	0.025	2.09
	II (3)	6302 ± 638	984 ± 274	15.53 ± 3.54	0.666 (1)	0.176 (1)	0.068 (1)	13.01 (1)
HAM	I (3)	7879 ± 732	1041 ± 39	13.5 ± 1.53	0.357 ± 0.044 (2)	0.095 ± 0.004 (2)	0.050 ± 0.006 (2)	2.67 ± 0.20 (2)
	II (3)	11,441 ± 1255	1018 ± 99	9.47 ± 1.93	0.860 (1)	0.169 (1)	0.126 (1)	4.85 (1)
ADD	I (5)	4410 ± 777^VL^	430 ± 60^VL^	10.68 ± 1.69	0.342 ± 0.014 (4)	0.150 ± 0.081 (4)	0.024 ± 0.015 (4)	2.27 ± 0.84 (4)
	II (4)	8065 ± 865^VL^	768 ± 76^VL^	9.93 ± 1.59	0.637 ± 0.100 (3)	0.214 ± 0.029 (3)	0.063 ± 0.026 (3)	4.02 ± 0.77 (3)
SOL	I (0)							
	II (2)	9693 ± 3580	799 ± 204	8.64 ± 1.09				

*Note:* Data are presented as mean ± SEM. Values between brackets: n. ^VL^: significantly different from VL at *p* ≤ 0.034.

Abbreviations: ADD, adductor magnus; BB, biceps brachii; FCSA, fiber cross‐sectional area; HAM: hamstring; Po, maximal isometric force; S, soleus; ST, specific tension; Vmax, maximal shortening velocity; VL, vastus lateralis; a/Po, indicator of the curvature of the force velocity relationship; Power_spec_, specific power.

For both bonobo and human, the FCSA of type II fibers was larger than that of corresponding type I fibers (*p* = 0.002; Table 2). In both species type II fibers had a higher Po than type I fibers (*p* < 0.001), but there was no significant difference in specific tension between fiber types. The bonobo fibers had a larger FCSA than the human fibers (*p* < 0.001), but there was no significant difference in Po between bonobo and human fibers, explaining the lower specific tension in bonobo than human fibers (*p* < 0.001).

**Table 2:** Contractile properties of type I and type II fibers from bonobos and humans.

	Type	FCSA (µm^2^)	Po (µN)	ST (N∙cm^‐2^)	Vmax (FL∙s^‐1^)	a/Po	Power (µW)	Power_spec_ (W ∙ L^‐1^)	Po/(Po∙Vmax)* 100,000
Bonobo	I (16)	7648 ± 790 (18)^a^	750 ± 60 (18)	10.7 ± 0.8 (18)^a^	0.295 ± 0.020^a^	0.119 ± 0.020^a^	0.028 ± 0.005	1.99 ± 0.28	12.57 ± 1.84^a^
	II (22)	9654 ± 593 (31)^a^^,^ b	1116 ± 70 (31)^b^	12.1 ± 0.7 (31)^a^	0.770 ± 0.032^a^^,^ b	0.195 ± 0.008^a^^,^ b	0.129 ± 0.018^a^^,^ b	7.73 ± 0.64^b^	13.55 ± 0.85^a^
Human	I (39)	5840 ± 378	791 ± 45	14.1 ± 0.5	0.373 ± 0.010	0.066 ± 0.003	0.021 ± 0.002	2.03 ± 0.12	7.19 ± 0.39
	II (46)	7037 ± 375^b^	966 ± 38^b^	13.7 ± 0.5	0.939 ± 0.026^b^	0.099 ± 0.003^b^	0.074 ± 0.005^b^	6.85 ± 0.36^b^	8.09 ± 0.52

*Note:* Data are presented as mean ± SEM. Values between bracket: n.

Abbreviations: FCSA, fiber cross‐sectional area; Po, maximal isometric force; ST, specific tension; Vmax, maximal shortening velocity; a/Po, indicator of the curvature of the force velocity relationship; Power_spec_, specific power.

^a^ significantly different from humans.

^b^ significantly different from type I at *p* < 0.05.

In both species the Vmax of type II fibers was higher than that of type I fibers (*p* < 0.001), and for each type, the Vmax was lower in bonobo than human fibers (*p* < 0.001). In both species a/Po was higher in type II than type I fibers (*p* < 0.001), and for each type, the bonobo fibers had a higher a/Po than the corresponding human fibers (*p* < 0.001); in type I and in type II fibers a/Po was 80% and 97% higher in bonobo, respectively. In both species, the maximal power of type II fibers was higher than that of type I fibers (*p* < 0.001), and the maximal power of bonobo type II fibers was 74% higher than that of human type II fibers (*p* < 0.001), but there were no significant differences in the maximal power of human and bonobo type I fibers. The specific power—power per unit fiber volume—was higher for type II than type I fibers (*p* < 0.001), but did not differ significantly between human and bonobo fibers.

**Figure 1:**
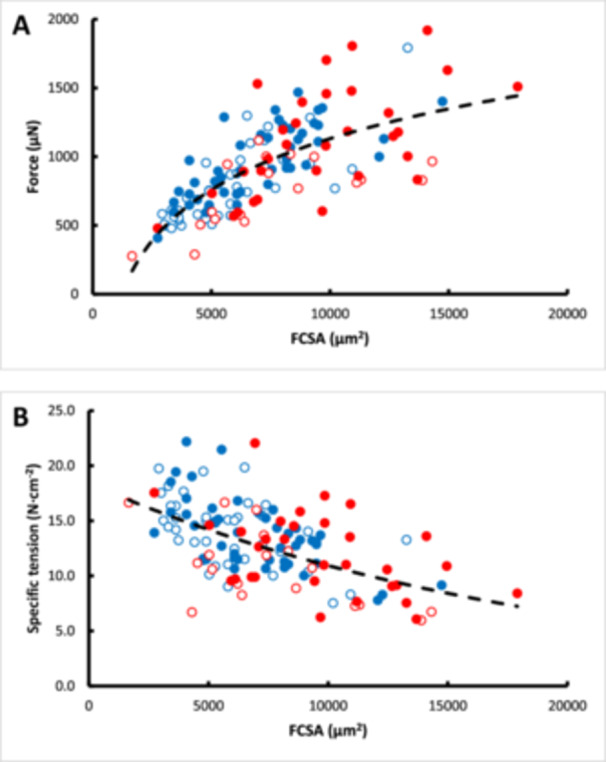
The relationship between (A) force (Po) and (B) specific tension with fiber cross‐sectional area (FCSA) in bonobo (red symbols) and human (blue symbols) fibers. Type I: open symbols; Type II: closed symbols. In panel (A) a logarithmic relationship between force and FCSA was the best fit (Po = 536·ln(FCSA)−381; R^2^ = 0.509; *p* < 0.001), and in panel (B) it was an exponential relationship (Specific tension = 18.4·e^‐5^·^10‐5^·^FCSA^; R^2^ = 0.314; *p* < 0.001) through all data pooled (dashed black line).

Po correlated with FCSA (linear: R^2^_adj_ = 0.472; *p* < 0.001; logarithmic, as shown in Figure 1A: R^2^ = 0.509; *p* < 0.001), which was increased by inclusion of fiber type (R^2^_adj_ = 0.508; *p* = 0.001) and slightly further with inclusion of species (R^2^_adj_ = 0.520; *p* = 0.039). The latter reflected the larger FCSA of bonobo than human fibers (Table 1). There was a negative relationship between specific tension and FCSA (linear: R^2^ = 0.304; *p* < 0.001; exponential in Figure 1B: R^2^ = 0.314; *p* < 0.001), which improved somewhat with inclusion of fiber type (R^2^ = 0.328; *p* = 0.020).

**Figure 2:**
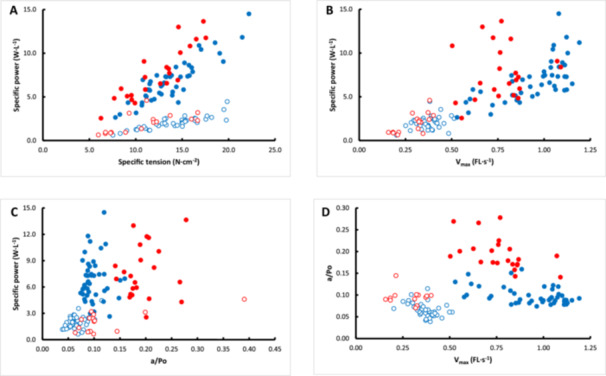
The relationship between specific power with (A) specific tension, (B) maximal shortening velocity (Vmax) and (C) a/Po and (D) relationship between a/Po and Vmax in bonobo (red symbols) and human (blue symbols) fibers. Type I: open symbols; Type II: closed symbols. For the description of the statistics, see text.

Figure 2A shows the relationship between specific power and specific tension. Stepwise regression shows that fiber type was related to specific power (R^2^_adj_ = 0.608; *p* < 0.001), with an improvement when specific tension (R^2^_adj_ = 0.796; *p* < 0.001) and a further improvement when species (R^2^_adj_ = 0.840; *p* < 0.001) was included. In the figure this is reflected by the higher specific power of type II than type I fibers, and a higher specific power of bonobo than human fibers for a given specific tension.

Figure 2B shows the relationship between specific power and Vmax (R^2^_adj_ = 0.633; *p* < 0.001), with an additional contribution of species (R^2^_adj_ = 0.685; *p* < 0.001), as seen as a higher specific power in type II bonobo than type II human fibers at a given Vmax.

Figure 2C shows the relationship between specific power and a/Po. Fiber type was the main determinant (R^2^_adj_ = 0.608; *p* < 0.001) which improved with addition of a/Po (R^2^_adj_ = 0.623; *p* < 0.016).

When including Vmax, specific tension and a/Po in a stepwise regression model for specific power of a muscle fiber, it appeared that Vmax was the main determinant of specific power (R^2^_adj_ = 0.633; *p* < 0.001), followed by a/Po (R^2^_adj_ = 0.735; *p* < 0.001), specific tension (R^2^_adj_ = 0.870; *p* < 0.001), species (R^2^_adj_ = 0.877; *p* = 0.002) and fiber type (R^2^_adj_ = 0.885; *p* = 0.002). The higher specific power in bonobo than human fibers for a given specific tension and Vmax was attributable to their lower curvature (higher a/Po, see Table 1).

Figure 2D shows that at a given Vmax bonobo fibers had generally a higher a/Po than human fibers. It also shows that in both human (R^2^ = 0.206; *p* = 0.002) and bonobo (R^2^ = 0.250; *p* = 0.018) type II fibers, and in human (R^2^ = 0.158; *p* = 0.012) but not bonobo (R^2^ = 0.206; *p* = 0.002) type I fibers there was an inverse relationship between a/Po and Vmax.

**Figure 3:**
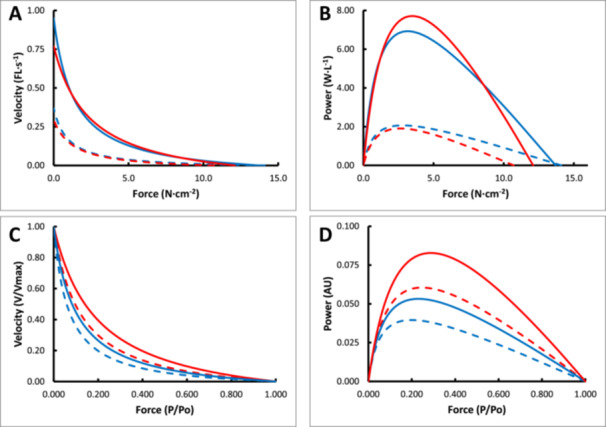
Illustration of the average (A) force‐velocity and (B) force‐power curves, and normalized (C) force‐velocity and (D) force‐power curves in bonobo (red curves) and human (blue curves) fibers. Type I: dashed lines; Type II: solid lines.

Figure 3A illustrates the average force‐velocity curves for the human (blue lines) and bonobo (red lines) type I (dashed lines) and type II (solid lines) fibers, using the data from Table 1. Figure 3B illustrates that despite the lower Vmax and specific tension in bonobo than human fibers there was no significant difference in the maximal power between bonobo and human fibers (Table 1). Figure 3C normalizes the force‐velocity relationship to Po and Vmax, showing indeed that the lower curvature (higher a/Po; Table 1) in bonobo than human fibers—Po and Vmax being the same—resulted in a 25% and 38% elevated maximal power in type I and type II fibers, respectively (Figure 3D), where the normalized force‐velocity profile of bonobo type I fibers even exceeded that of human type II fibers. This indicates that despite lower Vmax and specific tension in bonobo compared to human fibers, their similar specific power is attributable to this compensatory impact of the lower curvature (higher a/Po) in bonobo fibers on specific power.

## 4 Discussion

The main observation of the present study is that type II bonobo fibers could generate more power than human type II fibers. In line with our hypothesis, this was not only attributable to their larger fiber size, but also to the smaller curvature of the force‐velocity relationship (higher a/Po). The specific power (power per litre muscle volume) was, however, similar in bonobo and human fibers. Thus, the smaller curvature (higher a/Po or “shape factor”) of bonobo fibers was enough to offset the impact of the lower specific tension and maximal shortening velocity than human fibers on specific power. Therefore, the higher specific power of bonobo muscles must be—assuming the fiber type composition is similar to that in the chimpanzee—attributable to a higher proportion of fast fibers (O'Neill et al. 2017).

### 4.1 Force Generating Capacity

In contrast to the suggestion that the better jumping performance of the bonobo than humans is due to a larger specific tension (Scholz et al. 2006), we found a lower specific tension in bonobo fibers. Although this was not significant in an earlier study (O'Neill et al. 2017), their data also show a suggestion of a lower specific tension in chimpanzee fibers. However, the fibers of the bonobo were on average larger than those in humans. As the inverse relationship between specific tension and fiber size in humans (Gilliver et al. 2009) was similar in bonobos (Figure 1B), for a fiber of a given size there is no significant difference in the specific tension between bonobos and humans. Whatever the explanation for this inverse relationship between specific tension and fiber size, the “super strength” of the bonobo cannot be explained by a higher specific tension.

### 4.2 Maximal Shortening Velocity

The almost 4x higher specific power of type II fibers than type I fibers is largely attributable to the higher Vmax of type II than type I fibers (Bottinelli et al. 1996; Gilliver et al. 2009). Here we also found that in both bonobo and human fibers the Vmax and specific power were higher in type II than type I fibers. It appeared that Vmax was the prime determinant of the specific power of a muscle fiber, irrespective of species. At first glance this seems to support the idea that there are no intrinsic differences between the contractile properties of human and bonobo or chimpanzee fibers (O'Neill et al. 2017). Yet, we observed that bonobo fibers had a lower Vmax than human fibers. Part of the discrepancy may be related to the determination of the maximal shortening velocity, where (O'Neill et al. 2017) used the slack test (O'Neill *vs.* our data: I: 0.77 *vs.* 0.29; II: 2.75 *vs.* 0.77 fiber lengths·s^‐1^), while we derived it from fitting the force‐velocity data to the Hill equation. Even so, also in their study the average unloaded shortening velocities of type I and IIa fibers were, if anything, lower in chimpanzees than humans, except for one type IId fiber. Whether the maximal shortening velocity was lower or not, both observations confirm the earlier suggestion (O'Neill et al. 2017) that differences in Vmax cannot explain the better performance of bonobo and chimpanzee muscles.

### 4.3 a/Po

The curvature of the force‐velocity relationship (a/Po) has a significant impact on muscle power (Gilliver et al. 2009). In both humans and bonobos type II fibers had a higher a/Po than type I fibers, and as previously seen in rat type I (Gilliver et al. 2011b) and human type I and type II (Gilliver et al. 2009) fibers, also in bonobos a/Po was somewhat inversely related to Vmax. In addition, for a given Vmax bonobo fibers had in general a higher a/Po than human fibers. It is not clear what determines the curvature of the force‐velocity relationship, but it seems mechanisms that affect velocity will have an inverse effect on a/Po as reflected by a reduced Vmax and elevated a/Po after reducing the calcium concentration of the activating solution (Gilliver et al. 2011a), while the reverse was found in response to an elevated inorganic phosphate concentration (Degens and Jones 2020). These effects of a reduced calcium concentration and elevated inorganic phosphate concentrations may be mediated via alterations in the proportion of low‐force cross‐bridges. It is, however, unlikely that either factor explains the inter‐species difference, as the calcium and inorganic phosphate concentrations in the fiber are determined by their concentrations in the activating solution. Another possibility is that there are different posttranslational modifications between human and bonobo fibers where we have seen, for instance, that exposure to hydrogen peroxide induces a reduction in Vmax and specific tension without a significant change in a/Po (Gilliver et al. 2010).

Here we found that the contribution of a/Po to power is more important than specific tension. A lower curvature (higher a/Po) would comply with the idea that for the superior jump performance of the bonobo “*the force delivered at a certain shortening velocity must have been very high*” (Scholz et al. 2006). Other studies have not investigated this in chimpanzees or bonobos. However, one study did show higher a/Po values, without a difference in Vmax and specific tension, in muscle fibers from marmosets than those from humans (Plas et al. 2015) that would have contributed to the higher muscle specific power in those animals. Given that there was no significant difference in specific power between bonobo and human fibers, the higher a/Po must have been sufficient to offset the reduction in power due to the lower specific tension and Vmax. Although the higher a/Po did not result in a higher specific power, the implication is that at the same relative shortening velocity (in terms of FL·s^‐1^) bonobo fibers were indeed able to generate more force and power, which may have contributed to their better jump performance. Another factor is that chimpanzees (O'Neill et al. 2017) and bonobos have longer fibers, resulting in a faster shortening velocity in mm·s^‐1^, and hence power.

While a higher a/Po is beneficial for maximal power and force at a given shortening velocity, this comes at an energetic cost. Indeed, a classical study has shown that the large curvature (low a/Po) of the force‐velocity relationship in tortoise skeletal muscle was associated with a high energy efficiency (Woledge 1968). Interestingly, in rats with heart failure with preserved ejection fraction, the shortening velocity and specific tension were unaltered, but yet it could be deduced that the curvature of the force‐velocity relationship was increased (Espino‐Gonzalez et al. 2021), which was suggested to be an adaptation to the impaired oxygen delivery (Hendrickse and Degens 2021). Hence, the higher a/Po may be another factor that contributes to the low energy inefficiency in bipedal locomotion in chimpanzees (Sockol et al. 2007). We therefore suggest that the higher a/Po in the bonobo, yielding higher forces at a given shortening velocity (i) facilitates their climbing and jumping ability that require large forces at (ii) the expense of endurance, while in human muscles the opposite applies.

### 4.4 Limitation

The bonobo fibers were obtained from just one old female animal that died of heart failure and compared to fibers from young healthy men. While it has been reported that both heart failure (Miller et al. 2014) and ageing (D'Antona 2003; Degens et al. 1998; Larsson et al. 1997) may affect muscle fiber function negatively, other studies have shown that any effect of ageing on single fiber (Sundberg et al. 2025; Trappe et al. 2003) or heart failure on muscle contractile properties (Espino‐Gonzalez et al. 2021) were explicable by atrophy.

Another potential limitation is that bonobo fibers were derived from different muscles. However, it has been reported that “*Fibers belonging to the same type showed identical contractile parameters regardless of the muscle of origin*” (Harridge et al. 1996). Although there are errors in determination of the FCSA, we measured the cross‐sectional area while the fiber was suspended in the air, and it is a reasonable assumption—because of surface tension—that in that case the fiber assumes a circular circumference. In fact, we found that the measurement of the cross‐sectional area from the width while the fiber was suspended in the air was closest to the cross‐sectional area of fibers from the same muscles in histological sections (Degens and Larsson 2007). Whatever the error in the measurement of the FCSA, this applies to both the human and bonobo fibers. Finally, the experiments were performed at 15°C, far from the temperature at which muscles typically act. However, it is to be expected that the temperature sensitivity does not differ between bonobo and human muscle fibers. Given that the study largely corresponds with previous observations in chimpanzee (O'Neill et al. 2017) and marmoset (Plas et al. 2015), and both bonobo and human fiber contractile properties were done at the same time, under the same circumstances and with identical solutions, we expect that these limitations had a minor impact on the outcome of the study.

Future studies may also investigate the calcium sensitivity and rate of force development with the *K*_*tr*_ procedure.

## 5 Conclusion

Overall, a lower specific power in bonobo fibers expected from their lower specific tension and Vmax was apparently offset by an increase in a/Po so that human and bonobo fibers have a similar specific power. Given the greater power of type II than type I fibers, it is likely that the larger proportion of type II fibers in chimpanzee than human muscles is, as suggested previously (O'Neill et al. 2017), the main explanation for the “super strength” in bonobos.

## Data Availability

The data are available at https://doi.org/10.23634/MMU.00640738.
